# Indents

**Published:** 1907-04-15

**Authors:** 


### INDENTS.

☞ **Brief Articles of Technical or Scientific Value will receive notice and comment. Contributions invited.**

**Obtunding Sensitive Dentin.** After the dam is in position, and the cavity dried out as well as can be with cotton and spunk, dip a piece of spunk in carbolic acid and place in cavity: then heat a burnisher or ball-headed plugger and apply to spunk, gently at first and then with pressure, and repeat till all sensation is gone. Y. T. Coghlan, *Western Dental Journal.*

**Defective Enamel.** I have found beneficial results in cases of deficient enamel in children's teeth by filling the irregular cavities, or surfaces, with some sticky cement. In time this can be ground down somewhat, and the gentle irritation of the dental fibrils will cause a protective growth, which increases the resisting power of the dentine against decay.—R. R. Andrews, in *Journal.*

**Stanno-Percha.** Equal parts, by weight, of gutta-percha and sifted sponge tin are put in a mortar, the mortar placed in a sand bath and the ingredients thoroughly kneaded until a grayish violet mass is obtained. One-half is to be used soft for cavity lining. The other half is again kneaded with an equal amount of sponge tin, to be used for the body of the filling.—Dr. Arthur Scheuer, *Era.*

**Silk First—Then the Clamps.** I desire to call attention to the application of one or two ligatures in placing a clamp upon a tooth. One circle of floss silk will prevent the clamp from impinging upon the gum. If the tooth is cone shaped, two should be put on; one will prevent the clamp from moving upward, and the other from jumping off, and there will be saved the pain from impingement.—Dr. N. C. Register, *Brief.*

**Die Metal for Modeling Compound Impressions.** In cases where it is impossible to secure a plaster of Paris impression a die can be obtained from modeling compound by using the following die metal: Bismuth, 48 per cent.; cadmium, 13 per cent,; lead, 19 per cent.; tin, 20 per cent. This can also be poured into wet plaster of paris with little or no risk.—Dr. O. H. Simpson, *Dentist's Magazine.*

**Sterilizing Impression Trays and Impression.** Impression trays should be thoroughly washed and polished when the impression material has been removed. Impression material may be effectively sterilized without damage by placing it in a double saucepan with a lid having a hole in it for a thermometer and keeping it at a temperature of 160 degrees F. for an hour and a half.—J. H. Badcock, *British Dental Journal.*

**Conservation versus Practicability.** Dr. G. V. Black cites the case ot a small cavity in the mesial surface of a central incisor in the mouth of a young girl exceedingly sensitive to pain and asks, which should be considered first, the salvation of the tooth by a tedious and painful operation or the health of the patient? By all means adopt an expedient that is practicable and wait until the saving operation can be made with comfort and safety.

**Cut Solder Large.** Solder should be used in as large pieces as can be put on the work. One or two pieces the right size for a crown will give better results than twenty small ones. The reason for this is that oxide on a large piece is much less in proportion to bulk than the skin on a small piece, and the weight of the large piece will break the skin easier. Again, less borax is necessary, and the least borax used the better, provided you use enough.—Dr. C. C. Allen, *Western*.

**Petrogen.** Of late I have seemed to find benefit in directing patients, and I now do it in all cases, to rub on one of the combinations of iodine and oil, like Wyeth's "petrogen" into the gum, above the tooth, two or three times on the day following the filling of a root canal. These combinations do not stain and do not blister and are said to stimulate the phagocytes to increased endeavor to destroy the morbific bacteria.—C. P. Briggs, October 1906, *Journal*.

**Sensitive Dentin.** Some time ago Dr. Fossume spoke of the use of "Formalin" for controlling the sensitive spots at the necks of the teeth that are sometimes so troublesome. At that time I questioned its safety, but since trying the method I have found it so satisfactory that I wish to speak of it at this time. The "Formalin" should be used full strength and it must be kept away from the gum. A very short application is sufficient to control entirely a very large proportion of these cases.—H. W. Gillett, October 1906 *Journal*.

**Method of Setting an Inlay.** Instead of using direct pressure, as so many dentists do when they wedge an inlay to place, I put the inlay in position and draw a wide piece of tape over it, as I would in polishing a gold filling with a polisher. This keeps teetering the inlay from one end to the other, and if you keep going over it you will be sur prised how much closes the inlay will become seated than if you use any amount of direct pressure. Direct pressure on the inlay will not squeeze out the cement because the particles of cement rest on top of each other and cannot get away. I use a piece of tape twice the width of the inlay.—W. H. Taggart, *Dental Review*.

**Treatment of Sensitive Palates Prior to the Taking of Plaster Impressions.** In prosthetic work, when your patient has a tender palate and is bothered with retching, to stay such trouble while taking an impression, paint on palate a twenty per cent. solution of potassium bromid with a camel's-hair brush. or gargle 5 to 15 gr., or both. In extreme cases give 10 gr. at night, repeat after breakfast, and again, half an hour before impression is taken, give 10 to 15 gr. It greatly diminishes the sensitivity of a tender palate. —H. E. Davis, *Dental Era.*

**Removing Broken Pivots from Root-canals.** I have read a number of suggestions regarding the removal of broken pins of Logan crowns from root-canals, but am not aware that the present method has been previously published. I take a rose-head bur, follow the pin, and cut it out instead of the hard tissues around it. Platinum pins are soft, and I find no difficulty in cutting them out. I have never had any difficulty whatever in drilling a pin out, regardless of its length or of the nature of the root-canal.—B. B. Detwiler *in Cosmos.*

**Treating Putrescence in Roots.** Many methods have been proposed to render gases inert. I have found that a crystal of mono-chloro-acetic acid will do this. In all cases of putrescence within a pulp chamber or root canal, by first isolating the tooth and washing its interior carefully with copper sterilized water, then partially drying it, I place a crystal block of the acid in the center of the pulp chamber, and if there is not sufficient moisture, a drop of sterilized water is added. Within five minutes the tooth may be sealed hermetically with cement. Leave the tooth alone for a few days and open it under antiseptic precautions. You do not need to be changing the dressing every day or two. The worst case of putrescence will yield after two treatments at intervals of one week.—A. W. Harlan, in December *Review.*

**We are now a Profession.** Judge Taylor in the circuit court at St. Louis, Mo., has handed down a decision in which he declares that although the practice of dentistry may formerly not have been regarded as one of the learned professions it is so regarded today and takes rank with the practice of medicine and the law. He further states that a citizen has the right to engage in any business that is not prohibited by some special law, but not so with the learned professions. The case was the State of Missouri against the National Dental Parlors.—*American Journal.*

**Gold Inlay.** After preparing the cavity, taking the impression in compound, I fill the impression with Archite cement, which sets very quickly, as you all know. You can separate the impression and the cement very soon. Place a die so made into the swaging press and with a little piece of gold upon it swage a matrix, putting it back into the cavity for a little final burnishing, and then fill your matrix with 22-k. solder or plate. The whole is then put back into the cavity, the edges polished, and the inlay set. The entire operation can be done in about fifteen minutes.

**Pyorrhoea Treatment.** I wish to call your attention to a syringe that I have found useful in the treatment of cases of pyorrhoea alveolaris. About four years ago Dr. Eames suggested for the placing of treatments in pyorrhoea pockets, a syringe in which he used a vaseline mixture, with the idea that the effect would be more lasting. Later Dr. Wheeler talked about acetezone for this purpose. I found this very effective, but I disliked his means of getting it into place. I therefor was led to adopt Dr. Eames' idea so far as the syringe is concerned, mixing the acetezone with vaseline at the time of use. After mixing it must be used within fifteen or twenty minutes.—H. W. Gillett, in October 1906 *Journal.*

**Objection to Adrenalin Chlorid.** The well known liability of secondary hemorrhage after the use of adrenalin renders it unsatisfactory as a menstruum for cocain in pressure anæsthesia. I recently used it in removing the pulp from a central incisor and packed the canal with cotton saturated with an anæsthetic. At the next sitting I found the entire crown of a decidedly pinkish hue, showing a thorough infiltration of the tooth substance with hæmaglobin. The vigorous use of pyrozone practically restored the tooth to its natural color, though not entirely so.—A. J. Cotterell *in Brief.*

**Extracting Live Pulps.** When one has extracted the pulp down to its finest termination curling as it dies on the Donaldson broach, one is sometimes surprised on pushing up a cotton-wound broach to dry the canal, to get an expression of acute pain that makes one wonder whether by any chance, a bit of pulp remains, Persisting in the effort to dry the canal, the pain is gone, and one may vigorously prod without causing any sensation. I did not realize how acutely painful a little moisture could make a tooth until I had the experience in a tooth of my own.—C. P. Briggs, in October, 1906, *Journal.*

**Troubles of Various Dentists.** A dentist of Little Rock, Ark., gave himself up to the officers of Memphis, Tenn., his former home, on Jan. 2, to answer a charge of having embezzled $500 from the company for which he worked in Little Rock.—A dentist of York, Pa., was arrested on Dec. 12, and failing to give bail, was put in jail on complaint of his wife, who charged him with disturbing the peace by coming home when intoxicated and beating her.—A case against a Joliet (Ill.) dentist for disorderly conduct, was discharged. He was charged by two patients, a woman and her granddaughter, with having beaten them with a book.—A Milwaukee dentist was sent to the house of correction on Dec. 18, to serve a year, for abandonment of his wife and seven children.—A dentist of Muncie, Ind., was found guilty of larceny on Dec. 10, 1906, in an unusual case growing out of a political fight. In November, 1905, a question of eligibility of the election board arose, and the dentist, who was the clerk of the old board, refused to turn over the records and books to the clerk of the new board. The case was taken to the courts and has been in litigation ever since.—A San Francisco dentist is under arrest for the alleged embezzlement of $65 from the National Blue Cross Hospital Association.—A Cambridge (Ohio) dentist was arrested on Dec. 15, charged with having assaulted a young woman of the town.—A dentist of Macomb, Ill., brought suit Jan. 3, against a woman for $60 for work done. She claimed the work was not satisfactory. The decision was a compromise, the court ordering the dentist to make some change in the teeth and ordering the woman to pay in full.—A young woman of Kansas City, Mo., lost a suit in which she tried to recover $12 from a dentist because the teeth he had made for her did not "fill out her hollow cheeks," as she had wanted them to do.—A Council Bluffs (Iowa) dentist is suing the parents of his wife for the alienation of her affections. He claims that he aroused their enmity by refusing to become a member of the Latter Day Saints church, of which they were members.—A dentist of Lynn, Mass., has brought suit against the woman from whom he rents, alleging that the closing of the entrance to his office while repairs were being made to the building, injured his business.—A suit brought by a dental company of Cincinnati, Ohio, against two former employes, charging them with contempt of court for alleged violation of an order prohibiting them from using the mailing lists of the company, was dismissed.—A dentist of Pemberville, Ohio, is reported missing from his home under peculiar circumstances. He disappeared the day after Christmas, and a few days later his wife received a letter from him mailed at Toledo saying he never would return. He was clerk of the village. No shortage in his accounts or other reason for his departure has been discovered. *January*, 1907, *Digest.*

**A Method of Absolute Adhesion for Dentures.** By O. H. Simpson, D.D.S., Dodge City, Kan. About twenty-five years ago Dr. Speyer conceived the idea of increasing the mechanical adhesion of plates by checkering the inner surface of the plate. His method was to place tin foil over metal rolls with checkers in the foil, and by burnishing the foil over the model before vulcanizing, increasing the size of the plate the thickness of the foil. Checkers were cut in cameo, which became filled with soft tartar and food mucus. Thus the membrane became affected. I have undertaken to overcome this condition by making my checkers *in intaglio* instead of *in cameo.* This is done by pasting bobbinet on the impression. This decreases the size of the plate the thickness of the netting, while the old method of using tin foil increased the size of the plate the thickness of the foil.

Cut a strip of bobbinet wide enough to cover the buccal and labial surfaces of the impression on the upper surface of the flange; tack the netting on top of the impression to edge of the flange with a small quantity of warm wax, letting the strip fall down into the impression as a curtain or veil. Where there are sharp angles split the bobbinet with scissors, as you would a metal plate at the median line or other angles to facilitate the adaption After the net is adapted to the surface, varnish twice with good shellac varnish, being careful to paste the netting down at every point, as otherwise plaster of paris will run under the netting when the model is poured After the model is poured and opened take a sharp knife-blade or sharp instru nent and cut a groove on the palatal surface of the model, not over a quarter of an inch from the top of the ridge, extending over the heels of the plate to each condyle. This latter serves as a bead to which the plate may be finished at the heel. Drag a ball burnisher through this line, first made in the model by a sharp instrument, thus making a smooth groove or track. This will raise the bead of plate, making the suction cavity over the greatest area that possibly can be had. It relieves the pressure in the roof of the mouth, and does away mith the necessity of trimming the model or impression, as is usually the case. Take no precaution to guard against soft or hard spots in the mouth other than this. No paring is necessary. This bead will imbed itself in the soft tissue after a time and prevent the plate from being dislodged when biting on the front teeth. This checkering will increase the mechanical attachment at the flange, thereby making the greatest possible adhesion The bead should be made of depth in the model about the thickness of a sheet of blotting paper. It will not irritate the mouth, even though twice as thick. Impressions for dentures should be taken with stiff plaster of paris. When inserted in the mouth the impression tray should be rotated laterally, thus pushing the buccal and labial soft tissues out of the way, besides compressing the other soft tissue pads.—*Dental Brief.*

**Extracting Third Molars.** One morning a gentleman about thirty years ot age called on me and asked me to extract a lower third molar tooth for him. I had not known the gentleman a great while, but a short time previously I had inserted some fillings for him and at that time he had called my attention to an unerupted lower third molar which was troubling him slightly, telling me of the unsuccessful attempt of a dentist in another part of Kansas at extracting it, and of the refusal of other dentists to undertake the operation.

It did not look easy to me, but not anticipating failure, and knowing no good reason to refuse, I called a competent physician to give an anesthetic and proceeded. In this case a prolonged anesthesia seemed necessary, so we used chloroform.

Anesthesia was complete and muscles were completely relaxed. With mouth propped open and a pair or properly shaped alveolar forceps I took a good hold and tried to extract, but did not succeed. A second and third attempt ended likewise. I then tried an elevator and a pair of splitting forceps, with no better success with either, from the fact that the tooth was so deeply imbedded in the tissues that it seemed impossible to get under it, or between it and the second molar immediately in front and overhanging.

At this juncture, after consulting with the physician and knowing how greatly the patient desired the removal of the third molar, the removal of the second molar was decided upon. This was accomplished without great difficulty, although it had long, penetrating roots.

The field was now clear for the extraction of the objectionable tooth and the rest looked easy. A good firm hold was obtained deep on either side, but the tooth seemed as solid as the rock of Gibraltar, and did not shake with even a strenuous pull. Repeated efforts met with no better success, except at the last trial a portion of the crown was obtained.

Thinking I had accomplished some good for the patient in giving room for the tooth to erupt, and considering that other efforts at extracting likely to be equally fruitless, the patient was allowed to recover consciousness.

Swelling and inflamation persisted and about a month after operation a sequestrum, consisting of a piece of process about the length and breadth of a small thumb nail was removed. Smaller pieces were afterward removed from time to time, a slight swelling of the face on the outside and discharge of pus from the pocket on the inside continued and was still continuing four months after the operation, when the patient was in Kansas City and obtained an X-ray picture of the affected parts.

Today, seven months after the operation, the condition is practically as shown in the picture, which I have with me, the tooth gradually growing more prominent, or the gum shrinking, or both, but still apparently firm in the socket and a slight enlargement of the face on the outside.

In reciting this story I have perhaps wearied you with details, but considering its bearing and influence on me and my resolving to be more conservative in operating upon impacted third molars in the future, accounts to some extent perhaps for my treatment of another case more recently.

A lady presented herself for treatment with a similar lower molar showing an incipient alveolar abscess. She did not wish to have the tooth extracted, saying she was nervous, not strong, that she was seven months pregnant and that her home dentist where she formerly lived and two dentists in the city (both considered experts in extracting) all had refused to undertake the extracton.

At that time I drilled an opening to the pulp chamber, got a small flow of pus and blood from the root canal and dismissed the patient, who was feeling much relieved.

Subsequent treatment consisted of flushing with hot solutions of peroxide of hydrogen, under which she grew steadily worse.

I explained to her that the abscess was self limiting and that it could soon be lanced with little danger or difficulty and advised against extraction, especially as I had found upon further inquiry that she had a history of an attack of acute nephritis two years ago, had had several attacks of urethral colic, was not well; that it was a first pregnancy at twenty-eight years, and that difficult labor was anticipated.

Contrary to what I had expected the abscess did not terminate when it was lanced by a physician a week or so afterward, but continued to swell and to involve the parotid gland and surrounding tissues. A drainage was established and was maintained and conditions did not improve, Swelling increased, more tissues were involved, opening the mouth had become impossible, sleep and rest were obtained only by the use of opiates and it was in this condition I found her when I was again called to her home on January 6, 1907, seven weeks after my first treatment. Upon consultation with two physicians, and with their assistance, I extracted both second and third molars under anesthetic without great difficulty.—Dr. J. A. Thompson in Feb. *Western Journal.*

